# Evaluation of groundwater quality for drinking and irrigation purposes using proxy indices in the Gunabay watershed, Upper Blue Nile Basin, Ethiopia

**DOI:** 10.1016/j.heliyon.2023.e15263

**Published:** 2023-04-15

**Authors:** Asnakew Mulualem Tegegne, Tarun Kumar Lohani, Abunu Atlabachew Eshete

**Affiliations:** Water Technology Institute, Arba Minch University, Arba Minch, Ethiopia

**Keywords:** EWQI, Drinking water, Irrigation water indices, Wastewater management

## Abstract

Evaluation of groundwater potential and its quality assessment for drinking and irrigation has recently become a major concern, especially in developing countries due to various constraints. The primary aim of this study is to evaluate the quality of groundwater and establish whether they are safe for domestic and agricultural usage. 78 samples were collected during dry and wet seasons from 39 locations in the Gunabay district of the upper Blue Nile, Ethiopia. The following physicochemical parameters were evaluated successfully (T, pH, EC, TDS, Na^+^, K^+^, Ca^2+^, Mg^2+^, Fe, Cl^−^, F^−^, SO_4_^2−^, PO_4_^3−^, CO_3_^2−^, HCO_3_^−^, and NO_3_^−^-N). Then, Entropy Weight Water Quality Index (EWQI) and irrigation water quality indices (SAR, %Na, MAR, RSC, PS, KI, PI, and IWQI) were used to assess the distribution of groundwater quality in the study area. The Piper diagram used to characterize the groundwater types revealed that Ca–HCO_3_ is dominant in the area and rock-water interaction regulates the chemical characteristics of groundwater. Wilcox diagram was used to analyze the salinity level in the groundwater. The findings showed that the groundwater had higher nitrate levels relative to the permissible level of WHO standards due to excessive use of fertilizers in rural areas. Depending on the EWQI approach, the study area was categorized as excellent, good, and medium zones, covering 84.6%, 12.8%, and 2.6%, respectively. The results depict that high-quality drinking water was available in rural areas, n high to medium in the urban regions. The comparative irrigation water indices record 85% of water wells are suitable for irrigation, but some well sites are unsuitable due to higher salinity hazards and deep rock interaction. These integrated water quality indices were effective in validating drinking and irrigation water quality in the study area.

## 1 Introduction

Groundwater is extremely important for both rural and urban areas with limited surface water resources [1,2,3]. Approximately, 75% of the African population relies on groundwater for drinking and irrigation purposes [4]. This water supports socioeconomic development and human health improvement [5,6,3]. Groundwater is probably less contaminated from any hindrance created by climate change, droughts, and floods compared with surface water [7,8,9,6,3]. Generally, groundwater reservoir provides reliable, safe, and sustainable water for future generations if the source is judiciously managed. This requires considerable policies and laws, strategies and guidance, monitoring and management as well as investments and stakeholders [10]. However, the safety of groundwater (both shallow and deep groundwater sources) is potentially influenced by several factors, including the geological area, human and land use activities, environmental and climate conditions, and recharge (due to the interaction of surface and groundwater) [7,11,12,13,14,9].

On a global scale, the aquifers provide around 65% of the water for domestic uses, 20% for irrigation, 15% for industry, and many other activities, including hydropower generation [15,16]. At the Ethiopian national level, groundwater reservoirs have 80% of the water capacity for domestic use [17]. Ethiopia is one of the countries that use groundwater as a primary source [18]. Groundwater reservoirs provide a base flow that is the source of perennial rivers [19]. Around 3.5 billion people in the globe are deprived of sage and hygienic water supply [20]. The World Health Organization [21] stated that: "sufficient, safe, and accessible water must be available to all for sustainability.” On the other hand, the deterioration of groundwater has been increasing due to indirect and direct impacts of human activities and natural processes [22,23,24,25]. Groundwater quality evaluation at a reasonable time interval is obligatory to maintain its suitability for drinking and irrigation purposes [26,16]. Hence, it is a critical resource for current utilization and future preservation [27] for sustainable development.

The higher water needs and overexploitation have put immense stress on subsurface water availability and suitability, whereas on the contrary, groundwater contamination is becoming a worldwide challenge [28,29] which is more severe in rural communities and developing countries, including Ethiopia [17]. Subsurface water cannot be considered a suitable source for various uses due to industrialization, population growth, urbanization, hydrology, topography, climate, lithology, and anthropogenic activities [30,31,32,33,21,34]. Once the subsurface water is contaminated, it is difficult to treat the water quality in the groundwater reservoir [6,35]. Therefore, the determination of chemical properties for groundwater suitability (domestic, irrigation, and industrial uses) [36], regular groundwater monitoring, and superior management have become mandatory for sustainable water resource security [37,38]. Continuous groundwater monitoring is required for sustainable water resource development [39,40].

Several studies have been conducted on the evaluation of groundwater quality for drinking and irrigation [41,42] purposes in most parts of the Blue Nile basin. But unfortunately, the Gunabay watershed has been neglected so far. This area is mainly data-scarce in the upper part of the Blue Nile. To address this research gap, the current study is the first of its kind to assess the Abay Basin's groundwater quality status with a focus on its suitability for various uses. The study aims to investigate the current status of groundwater quality (levels of contamination), and characterize groundwater types to examine the suitability of groundwater quality for drinking and irrigation purposes. Besides, this study could help researchers, decision-makers, policymakers, and planners to create an advanced approach to ensure pure water supply and decide on the most effective groundwater management techniques in Ethiopia. Since identifying the origins and sources of poor groundwater quality and health risk problems can minimize the potential risk to public health due to groundwater deterioration [43].

## 2 Study area

### 2.1 Location of the study area

**Fig. 1:**
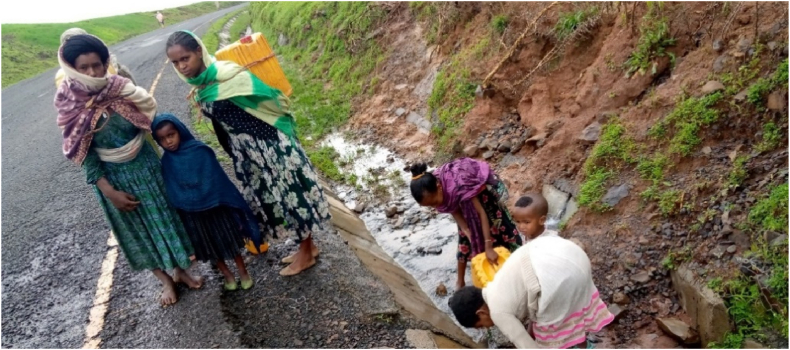
Untreated water access due to water scarcity near Andabet Town.

**Fig. 2:**
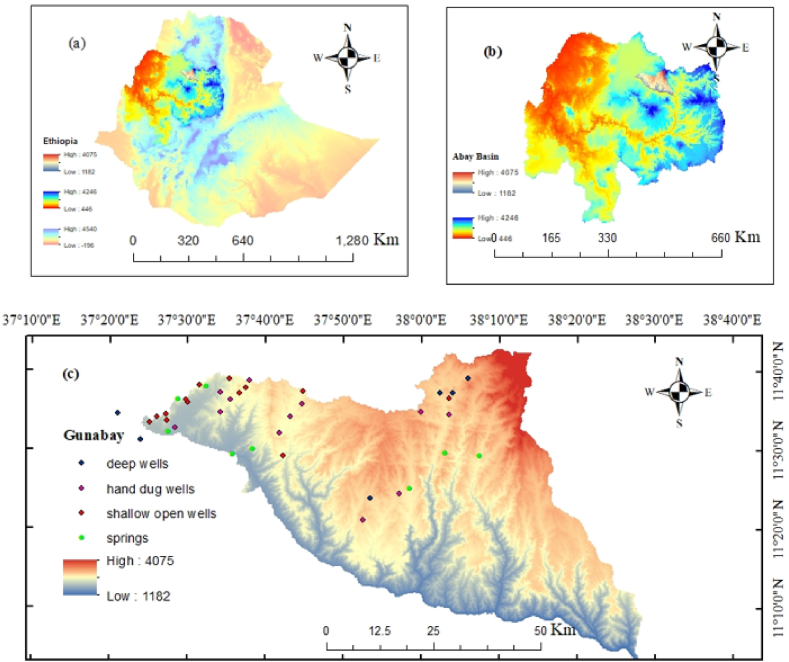
Map of the study area (a) Ethiopia (b) Abay Basin (c) Gunabay Watershed.

The study area is located in the northwest part of Ethiopia (Amhara National Regional State). Geographically, the area lies within the latitudes of 11°00′20″ N to 11°45′46″ N and longitudes of 37°23′06″ E to 38°28′24″ E. Gunabay is one of the watersheds of the upper Blue Nile basin (Fig. 2 a, b and c), with a total area of 3920.25 km^2^. Groundwater is the primary source of water for households and agricultural activities, yet Gunabay is not well known for its groundwater potential and quality aspects. The southeast part of the area has no adequate access to water for drinking purposes. There is no clean water available, particularly in the nearby towns of Andabet and Jaragedo, the community relies on runoff water which is neither safe nor hygienic (Fig. 1).

### 2.2 Geological setup

This study is occupied by Quaternary and tertiary rocks with thicknesses of up to 3000 m covering more than half of the country. The early quaternary and tertiary volcanoes are concentrated in the study area [44]. As shown in Table 1, slightly fractured and weathered basalt has a 33.3 m thickness and the top layer is covered with black cotton soil with a small 3 m thickness.

**Table 1:** Vertical distribution of geological setup of Dirkuat deep well site.

Drilled depth (m)		Lithologic Description	Thickness	Remarks
0.0–3.0		Top black cotton soil	3.0	
3.0–12.2		Gravel and boulder	9.2	
12.2–33.6		Highly fractured and weathered basalt	21.4	
33.6–54.5		Slightly fractured and weathered basalt	20.9	
54.5–58.0		Decomposed trachitic basalt	3.5	
58.0–64.1		Moderately weathered and fractured scoriaceous basalt	6.1	
64.1–70.2		Massive basalt	6.1	
70.2–76.3		Slightly fractured and weathered basalt interpolated with scoria	6.1	
76.3–109.5		Slightly fractured and weathered basalt	33.3	
109.5–119.0		Moderately fractured and weathered trachitic basalt	9.5	
119.0–128.1		Massive basalt	9.2	Major aquifer
128.1–148.5		Moderately fractured and weathered basalt	20.4	
148.5–152.5		Slightly fractured basalt	4.1	
152.5–155.6		Highly fractured and weathered basalt	3.1	
155.6–161.7		Massive basalt	6.1	
161.7–180.0		Highly fractured and weathered vesicular scoriaceous basalt	18.3	Minor aquifer
180.0–186.1		Moderately fractured and porous scoriaceous basalt	6.1	
186.1–204.4		Moderately fractured and weathered basalt	18.3	
204.4–210.5		Moderately fractured scoriaceous basalt	6.1	
210.5–216.6		Massive basalt	6.1	
216.6–241.1		Highly to moderately fractured and weathered scoriaceous basalt	24.4	Major aquifer
241.0–247.1		Massive basalt	6.1	
247.1–253.2		Moderately fractured and weathered basalt	6.1	
253.2–268.0		Massive basalt	14.9	

### 2.3 Land cover/uses

Land use (land cover) is one most influencing factor for the spatiotemporal variability of groundwater quality [45]. The dominant land cover in the study area was crops land (human-planted cereals) and shrub land (shrubs, bushes, and tufts of grass, savannas with sparse grasses or plants), which covers 57.04% and 36.16% respectively. Open water, forest, grassland, bare land, and settlement cover 6.8% of the total area. The land cover dataset is extracted from land cover databases of High-Resolution Maps (10:10) (https://livingatlas.arcgis.com/landcover).

## 3 Materials and methods

**Fig. 3:**
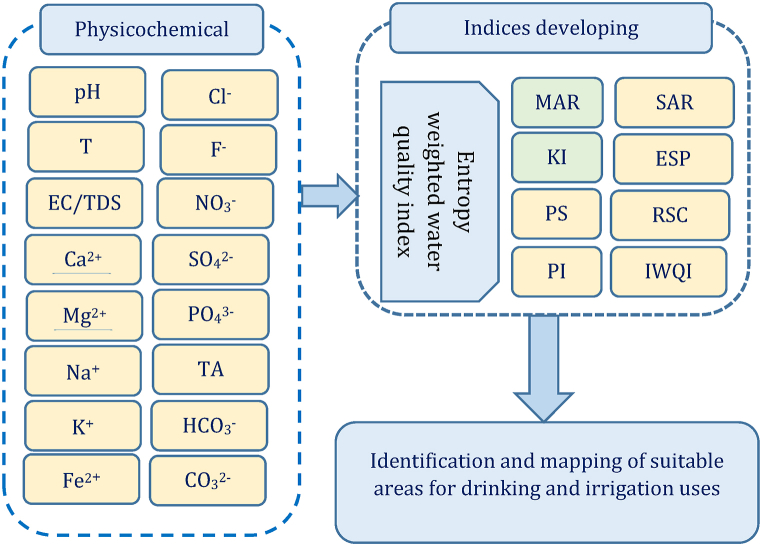
Methodological framework.

Constructing physicochemical parameters, developing drinking and irrigation water indices, and mapping groundwater quality potentiality zones of the study were the three research domains that were considered primarily. Matlab, Arc GIS tools, and AquaChem software were used for processing, and EWQI, SAR, RSC, ESP, MAR, KR, PS, PI, and IWQI were the indices calculated in detail (Fig. 3).

### 3.1 Data collection and analysis

In this study, different materials have been used to achieve the defined objectives. The following materials were used in the field: a Garmin GPS H72, a Micro Multi 800, and a water sample container (an icebox). The field samples were taken for two seasons, April 2022 (dry season) and August 2022 (wet season) and the laboratory tests were evaluated using the Palintest photometer 800 and flame atomic absorption spectroscopy.

#### 3.1.1 Sampling of groundwater

A systematic sampling technique was implemented to determine sampling sizes in the study area. 39 sampling sites were selected after considering the topographic setup (high land, escarpment, and lowland), geological setup, road accessibility, water well availability, literature reviews, and financial capacity. Therefore, 78 samples were collected with standard instruments from 8 springs (GW3, GW4, GW8, GW12, GW14, GW36, GW37, and GW38), 13 open wells (GW5, GW6, GW7, GW9, GW11, GW13, GW15, GW16, GW18, GW22, GW24, GW27, and GW31) and 12 hand-dug wells (GW10, GW17, GW19, GW20, GW21, GW23, GW25, GW26, GW32, GW33, GW34, and GW39), and 6 deep wells (GW1, GW2, GW28, GW29, GW30, and GW35). All shallow and deep wells have a depth of 6m–210 m. The basic parameters such as temperature (T⁰C), conductivity (EC), total dissolved solids (TDS), and pH were evaluated in the field using a micro multimeter. Using a pump to sample deep groundwater well is much more effective and the obtained sample is representative of deep aquifer water when using a pump [46]. This helps in removing the stagnant/polluted water from the wells. Polyethylene bottles of half-liter capacity were used to store sampled water. All sample bottles were stored in ice-packed coolers immediately after collection. The temperature of all stored samples was maintained at 0–4 °C until analyses were conducted.

#### 3.1.2 Major and minor physicochemical parameters

The total number of parameters was determined based on the literature review report, the cost of the laboratory tests, and the availability of laboratory equipment around the study area. Therefore, sixteen physicochemical parameters such as temperature (T⁰C), electric conductivity (EC), total dissolved solids (TDS), pH, sodium concentration (Na^+^), potassium concentration (K^+^), calcium concentration (Ca^2+^), magnesium concentration (Mg^2+^), Iron (Fe^2+^), chloride concentration (Cl^−^), fluoride concentration (F^−^), sulfate concentration (SO_4_^2−^), phosphate concentration (PO_4_^3−^), carbonate (CO_3_^2−^) and bicarbonate concentration (HCO_3_^−^), and nitrate concentration (NO_3_^−^-N) have been determined by standard procedures.

#### 3.1.3 Laboratory methods

A Palintest photometer was used for chemical parameters and flame atomic absorption spectroscopy (AAS) to measure sodium concentration. The laboratory analysis of chemical parameters has been analyzed at Abay Basin Authority, Amhara Design and Supervision Works Enterprise Laboratory Service, and ORDA, Ethiopia.

#### 3.1.4 Data accuracy assessment

The groundwater sampling data reliability has been checked using ionic balance error (IBE) or reaction error (RE), for good data representation. The results revealed that RE values are within ±5%. The accuracy of the chemical results was determined using equation (1) [42,24].

(1) $$IBE=\frac{\left(\sum Cation-\sum Anion\right)}{\left(\sum Cation+\sum Anion\right)}\;x\;100$$

#### 3.1.5 Water quality standards

To check the quality of groundwater for drinking, physicochemical parameters were evaluated and compared with the maximum permissible limit set by WHO [21].

### 3.2 Analysis of index for drinking purpose

Estimation of groundwater quality techniques has been developed and improved by different methods to get high performance and effective results. Recently, Entropy weighted water quality index (EWQI) has become the most acceptable approach for drinking water [34]. This is a reliable tool to evaluate overall quality for drinking purposes from the individual groundwater quality parameters [15,47]. In this study, the entropy-based groundwater quality index has been applied to evaluate the suitability of groundwater intentionally for drinking.

#### 3.2.1 Entropy weighted water quality index (EWQI)

The main aim of the water quality index is to characterize the overall quality of groundwater. According to the reports of numerous researchers, different water quality indices have been used for domestic groundwater usage [48]. Yet, the available approaches are makeshift by newly accurate techniques in apprehending the goal of representing the water quality. The entropy-weighted water quality Index (EWQI) is one of the most competent methods, which uses the entropy model to assign weights [49,39]. The approach of evaluating entropy weighted was analyzed in five steps, when m water samples (i = 1, 2 …, m) and each sample is analyzed for ‘‘n’’ quality parameters (j = 1, 2 …, n), according to observed data [50]. The first step is constructed through matrix X (equation (2)).

(2) $$X=\left(\begin{array}{cccc} x_{11} & x_{12} & . & x_{1n}\\ x_{21} & x_{22} & . & x_{2n}\\ . & . & . & .\\ x_{m1} & x_{m2} & . & x_{mn} \end{array}\right)$$

Where, X represents the initial matrix value of all parameters, m represents the total number of water samples and n represents the number of hydrochemical parameters.

In the second step, the initial process has to be standardized to remove the influences of dimensions and magnitude. Therefore, the standardized process was evaluated using equation (3), while the normalized process was carried out using equation (4).

(3) $$yij=\frac{x_{ij}-x_{ij\left(\min\right)}}{x_{ij}\left(\max\right)-x_{ij\left(\min\right)}}\;efficiency\;type$$

Where, xij is the initial matrix; (xij) min and (xij) max are the minimum and maximum values of the hadrochemical parameters of the samples, respectively.

(4) $$Y=\left(\begin{array}{cccc} y_{11} & y_{12} & . & y_{1n}\\ y_{21} & y_{22} & . & y_{2n}\\ . & . & . & .\\ y_{m1} & y_{m2} & . & y_{mn} \end{array}\right)$$

In the third step, the entropy “ej” and entropy weight “wj” have been computed by equations 5- 7.

(5) $$Pij=\frac{\left(1+yij\right)}{\sum_{i=1}^{m}\left(1+yij\right)}$$

This is entropy information, which can be calculated as in equation (6).

(6) $$ej=-\frac{1}{lnm}\sum_{i=1}^{m}PijlnPij$$

The entropy weight (wj) can be calculated in equation (7).

(7) $$wj=\frac{\left(1-ej\right)}{\sum_{i=1}^{m}\left(1-ej\right)}$$

In the fourth step, the quality rating scale (qi) has been calculated in equation (8).

(8) $$qi=\frac{Cj}{Sj}100$$

Cj is the concentration of water quality parameter j (mg/L), and Sj is the maximum permissible limits of drinking water by modified guideline of WHO [51] of parameter j (mg/L).

In the last step, the EWQI has been calculated using equation (9).

(9) EWQI=wiqi

Finally, the groundwater quality has been classified based on EWQI values as excellent (<25), good (25–50), medium (50–100), poor (100–150), and extremely poor (>150) for drinking uses [49,50].

### 3.3 Analysis of induces for irrigation purposes

The quality of groundwater may vary in space and time due to the extraction of groundwater, aquifer recharge, and the intensity of rainfall. Therefore, it is important to validate the quality of irrigation water [52], since, toxicity affects sensitive crops, salinity affects crop water availability, permeability affects soil infiltration rate, and miscellaneous effects susceptible crops [53,54].

The suitability of groundwater for irrigation purposes is mainly governed by SAR (Sodium adsorption ratio), %Na (Sodium percentage) or ESP (Exchangeable sodium percentage), PS (Potential salinity), MAR (magnesium adsorption ratio), RSC (residual sodium carbonate), Kelley index (KI), PI (permeability index) [55] and IWQI (Irrigation water quality index) [56,57]. However, as suggested by many agencies and organizations, suitability for this purpose relies on numerous indexes and parameters [58].

#### 3.3.1 Sodium adsorption ratio (SAR)

The initial concept of SAR was proposed by Richards [59], which is used for the detection of sodium hazards in the soil. It is vital for agricultural water classification, since the reaction of sodium and soil, forms sodium hazards. SAR can be calculated using equation (10) [60].

(10) $$SAR=\frac{\left({Na}^{+}\right)}{\sqrt{\frac{{Ca}^{2+}+{Mg}^{2+}}{2}}}$$

SAR can be classified as excellent quality for values of low sodium water (<10), good quality for values of sodium water (10–18), doubtful/poor quality for values of sodium water (18–26) and unsuitable quality for values of very high sodium water (>26) [15]. A value less than 10 increases the hydraulic conductivity of the soil texture and intensifies irrigation efficiency [58,55,50].

#### 3.3.2 Exchangeable sodium percentage (ESP)

The Na concentration in irrigation water is the result of the exchange of Ca and Mg concentrations, and the process reduces soil permeability. Sodium concentration (% Na) is an important approach for determining the quality of groundwater for agricultural activities. %Na (sodium percentage) or % ESP (exchangeable sodium percentage) can be computed by equation (11) [54].

(11) $$\%Na\;or\;ESP=\frac{{Na}^{+}+K^{+}}{{Ca}^{2+}+{Na}^{+}+{Mg}^{2+}+K^{+}}x\;100$$

Na percentage can be classified as excellent quality for values (<20), good quality for values (20–40), medium quality for values (40–60), doubtful/poor quality for values (60–80), and unsuitable quality for values (80–100) [15,54].

#### 3.3.3 Potential salinity (PS)

The potential of salinity in irrigation water is measured by electrical conductivity (EC), which is presented in microsemes per centimeter. Salinity hazards can be classified based on the EC (μS/cm) results: (very high, no detrimental effects for all crops) for the values (<750), (high, may have some detrimental effects for sensitive crops) for the values (750–1500), (moderate, may have adverse effects for many crops) for the values (1500–3000), (severe, not suitable for all crops) for the values (3000–7500) [55,52].

#### 3.3.4 Residual sodium carbonate (RSC)

It is a vital indicator of deep water in the confined aquifer with a higher amount of bicarbonate concentration (equation (12)) [61].

(12) $$RSC=\left({HCO3}^{2-}+{CO3}^{2-}\right)-\left({Ca}^{2+}+{Mg}^{2+}\right)$$

Therefore, RSC can be classified into three classes; good quality for values (<1.25), poor quality for values (1.25–2.5), and unsuitable quality for values (>2.5) [15].

#### 3.3.5 Magnesium adsorption ratio (MAR)

(13) $$MAR=\frac{{Mg}^{2+}}{{Ca}^{2+}+{Mg}^{2+}}\;100$$

MAR can be classified as suitable for irrigation for values (<50) and unsuitable for values (>50) (equation (13)) [15].

#### 3.3.6 Kelley index (KI)

Kelley's ratio (KR) is known as Kelley index, which was suggested by Kelley [62] for irrigation water quality analysis (equation (14)).

(14) $$KI=\frac{{Na}^{+}}{{Ca}^{2+}+{Mg}^{2+}}$$

Water is classified into three types based on the Kelly index. If the Kelly index value is less than one, the water is suitable for irrigation. Water is marginally suitable for irrigation if the Kelly index is between 1 and 2, and water is unsuitable if the Kelly index is greater than 2 [30].

#### 3.3.7 Permeability index (PI)

The permeability index (PI) is one of the most important indicators of groundwater suitability for agricultural activities. It is a measure of the ability of soil to move water (permeability). It correlates sodium, calcium, magnesium, and bicarbonate concentrations in soil, which are influenced by long-term irrigation practices (equation (15)).

(15) $$PI=\frac{{Na}^{+}+\sqrt{{HCO3}^{-}}}{{{Na}^{+}+Ca}^{2+}+{Mg}^{2+}}\;x100$$

PI values can be classified into three categories based on its suitability for irrigation: groundwater is suitable for irrigation (>75%), groundwater is good for irrigation (25–75%), and groundwater is unsuitable for irrigation (<25%) [54,9].

#### 3.3.8 Irrigation water quality index (IWQI)

It combines multiple indices and expresses irrigated agriculture water quality as a single value. Several authors have recommended using IWQI to evaluate irrigation water quality because it provides more reliable results [63]. IWQI established a clear grading system for irrigation water quality based on its effects on irrigated soil and plant toxicity (equation (16)). Electrical conductivity (EC), sodium adsorption ratio (SAR), sodium ion concentration (Na^+^), chloride ion concentration (Cl^−^), and bicarbonate ion concentration (HCO_3_^−^) are the major parameters to evaluate irrigation water quality [54].

(16) $$qi=qmax-\frac{\left(X_{ij}-Xinf\right)qimap}{X_{imap}}$$

Where qmax is the upper value of the corresponding class of qi

X_ij_ denotes the data points of the parameters (observed value of each parameter)

X_inf_ refers to the lower limit of the class to which the observed parameter belongs.

q_imap_ refers the class amplitude for qi classes

X_imap_ corresponds to class amplitude to which the parameter belongs.

(17) $$IWQI=\sum_{i=1}^{m}qiwi$$

Where, m is the number of parameters considered (qi) is a water quality measurement parameters values and (wi) is the weight of each parameter (equation (17), Table 2).

**Table 2:** Representative relative weights of IWQI parameters [64].

Parameters	wi
EC	0.211
SAR	0.189
Na^+^	0.204
HCO_3_^−^	0.202
Cl^−^	0.194

It can be classified into five categories as follows; no restriction (85–100), low restriction (70–85), moderate restriction (55–70), high restriction (40–55), and severe restriction (0-40). It can be visualized and evaluated for irrigation purposes using GIS for the parameters of the irrigation water quality index [54].

#### 3.3.9 Graphical representation of water irrigation uses

The much-accepted Wilcox diagram was used to evaluate groundwater quality for irrigation purposes. The vertical and horizontal axes of the diagram are SAR and electric conductivity (EC, μS/cm) which ranges from 0 to 40 and 100–12000, respectively [54,65].

## 4 Results and discussions

### 4.1 Physicochemical parameters and its seasonal variation

Evaluation of groundwater quality was conducted by considering proxy indices in this research. An entropy-weighted water quality index and graphical representation have been used to validate the water quality for drinking use in both seasons. In the same way, optimal irrigation water quality indices such as SAR, % Na (ESP), PS, RSC, MAR, KR, PI, IWQI, and graphical representation were also applied. The descriptive statistics of physicochemical values at 39 locations of Gunabay watershed during dry and wet seasons are given in Table 3. They revealed that, the variability of chemistry in the groundwater datasets in the area.

**Table 3:** Descriptive statistical values for measured physicochemical parameters during the two seasons.

Parameters	Minimum		Maximum		Standard deviation		WHO limit
	Dry	Wet	Dry	Wet	Dry	Wet	
T(⁰C)	21	17.6	31.1	20.4	2.08	0.82	–
pH	6.07	6.08	8.89	8.05	0.51	0.45	6.5–8.5
EC (μS/cm)	159.2	191	1602.0	1090	267.19	237.67	1000
TDS (mg/L)	101.88	122.24	1025.28	697.6	171	152.12	500–700
Ca^2+^ (mg/L)	5.0	6.0	60.0	62.0	12.33	12.6	75
Mg^2+^ (mg/L)	0	0	38.06	40.0	7.67	7.96	150
Na^+^ (mg/L)	0.90	0.92	186.0	185.92	32.51	32.54	200
K^+^ (mg/L)	0	0	3.23	3.4	0.98	1.05	–
Fe^2+^ (mg/L)	0	0	0.15	0.24	0.039	0.056	0.3
Cl^−^ (mg/L)	0	0	105.0	105.0	21.56	21.50	250
F^−^ (mg/L)	0	0	1.23	1.25	0.28	0.29	1.5
NO_3_^−^-N (mg/L)	0.7	0.74	31.6	33.2	9.12	9.68	11.36
SO_4_^2−^ (mg/L)	0	0	13.95	14.0	2.95	3.01	200
PO_4_^3−^ (mg/L)	0	0	2.68	2.80	0.49	0.5	250
TA (mg/L)	15.0	15.0	365.0	365.0	75.22	75.88	500
HCO_3_^−^ (mg/L)	15.0	15.0	390.0	395.0	80.9	81.72	150
CO_3_^2−^ (mg/L)	10.0	10.0	185.0	200.0	38.64	40.9	–

**Fig. 4:**
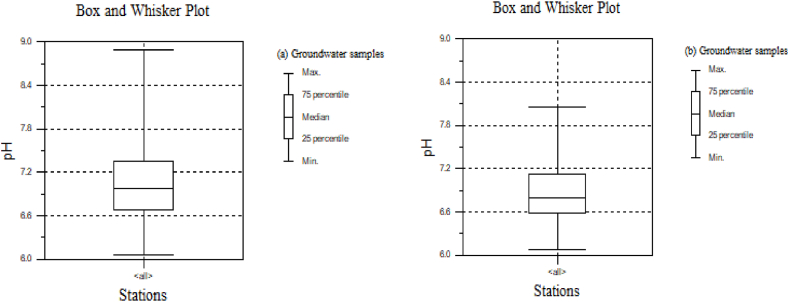
Seasonal pH variability (a) dry season and (b) wet season.

The seasonal variation of each physicochemical parameter is not significant in confined aquifers and deep groundwater wells. A slight variation had been observed in the study area. However, the seasonal variability in the two seasons is high for T(⁰C), EC (μS/cm), and TDS (Table 3). Unprotected springs and shallow wells have high NO_3_^−^-N levels. Due to extensive use of fertilizers, the concentration of nitrate in some shallow wells was above the maximum permissible standard limit [51]. The source of high nitrate concentrations in the groundwater is frequently attributed to anthropogenic activity (domestic waste and agricultural) due to lack of spring protection or wellhead [66]. The result shows that 5% of the samples were acidic in the dry season, whereas 13% of the total samples were acidic in the wet season. Only 2% of the total sample was basic, and more than 74% of the pH values decreased in the wet season (Fig. 4 a and b). This indicates that the locations of groundwater samples may become acidic in the future.

**Fig. 5:**
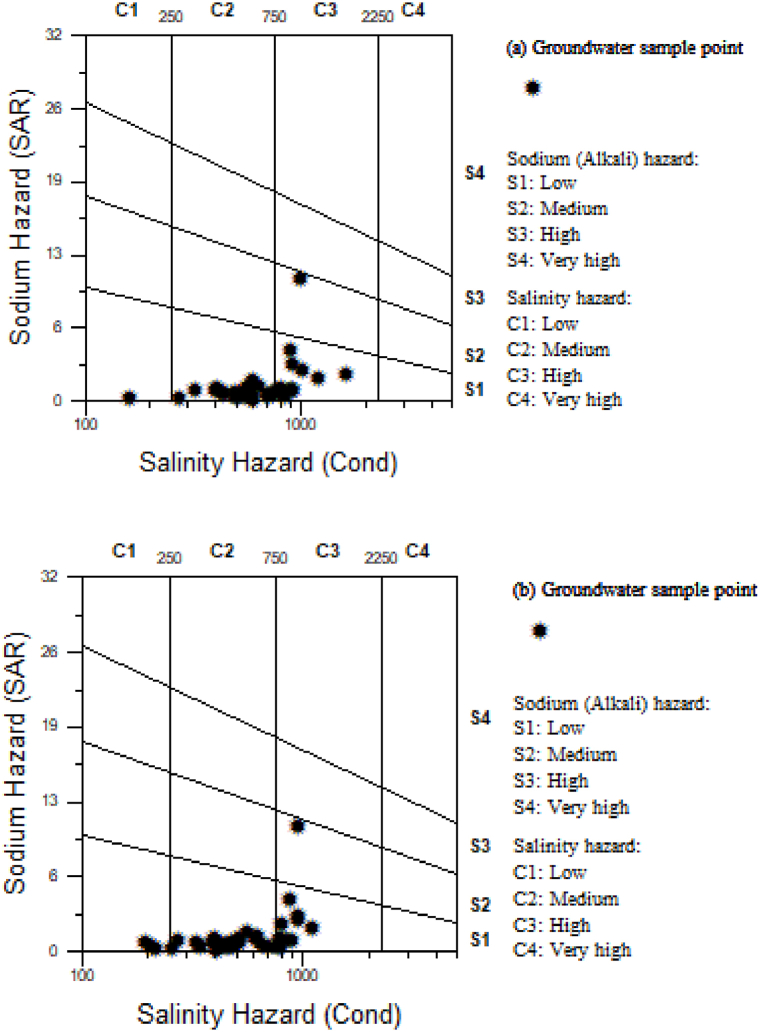
Seasonal variability of salinity effect (a) dry season and (b) wet season.

Moreover, 83% of the TDS values were higher in the dry season than the wet season. Arbgebya, Este, and Wanka well sites are the most exception sampling aquifers, where saline water intrusion makes groundwater unsuitable for irrigation purposes. Especially, Wanka well has medium sodium hazard (class: S2) and high salinity hazard (class: C3), which indicates the unsuitability of internal rock-water interaction for plant growth (Fig. 5 a and b). Therefore, for better irrigation purposes, such soil primarily needs efficient drainage patterns and good permeability.

### 4.2 Hydrogeochemical analysis

**Fig. 6:**
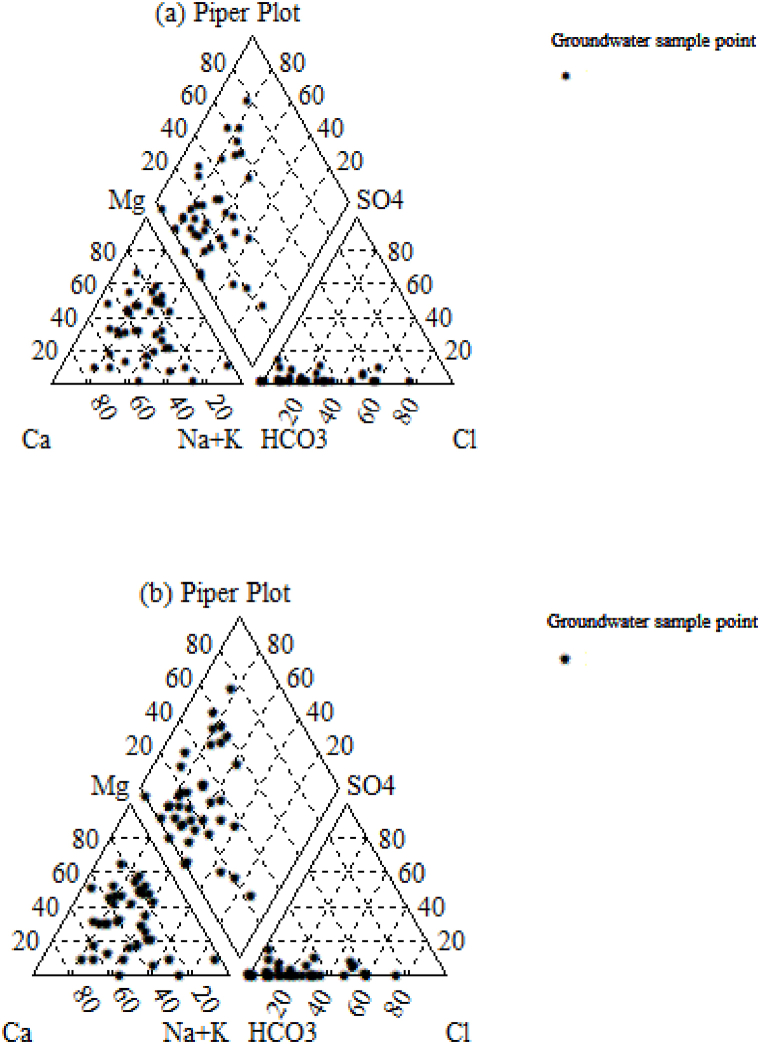
Type of groundwater based on the graphical representation of piper diagram (a) dry and (b) wet seasons.

Classification of water type and its characterization of hydrogeochemical processes were represented using a piper diagram that identifies the controlling factors of water chemistry [67,49,68]. Three water types accounted for 69% of the samples: Ca–HCO_3_ (left quadrant), Ca–Cl (18% of total samples), and Na–HCO_3_ (13% of total samples). As shown in Figure (6 a and b), most results revealed that groundwater in the study area is fresh and recharged. Around 18% of the samples indicated cement pollution or reverse ion exchange water. The remaining water samples are base-exchanged water, which implies a deep aquifer.

### 4.3 Gibbs plot

Groundwater chemistry can be controlled by physical properties of aquifer lithology (Table 1), weather condition, and bedrock mineralogy, which represents geochemical process of the rainfall, rock weathering, evaporation and precipitation-evaporation dominance [69,14].

**Fig. 7:**
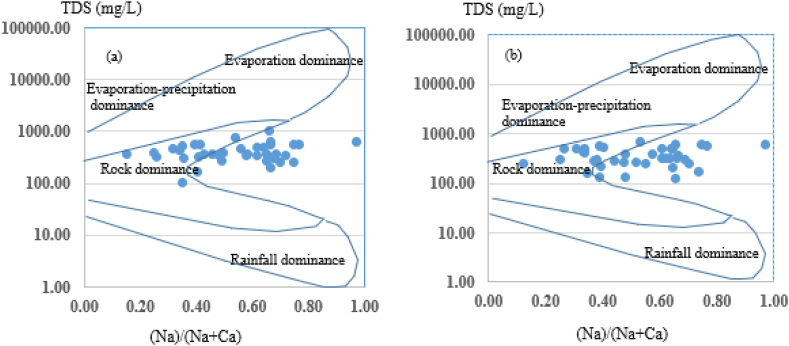
Gibbs plot for representation of geochemical process and groundwater chemistry in the study area (a) dry season and (b) wet season.

As indicated by Gibbs's diagram, the water quality in the study area is mainly influenced by rock weathering process (Fig. 7 a and b). This type of groundwater is showing the presence of water rock interaction due to carbonate and silicate rock weathering [14].

### 4.4 Water for drinking purpose

The water quality assurance, control, and water safety plans are responsible for drinking water supply agencies [51]. Therefore, this research provides a detailed investigation of water quality evidence for the communities and policymakers.

#### 4.4.1 Entropy-weighted water quality index

**Fig. 8:**
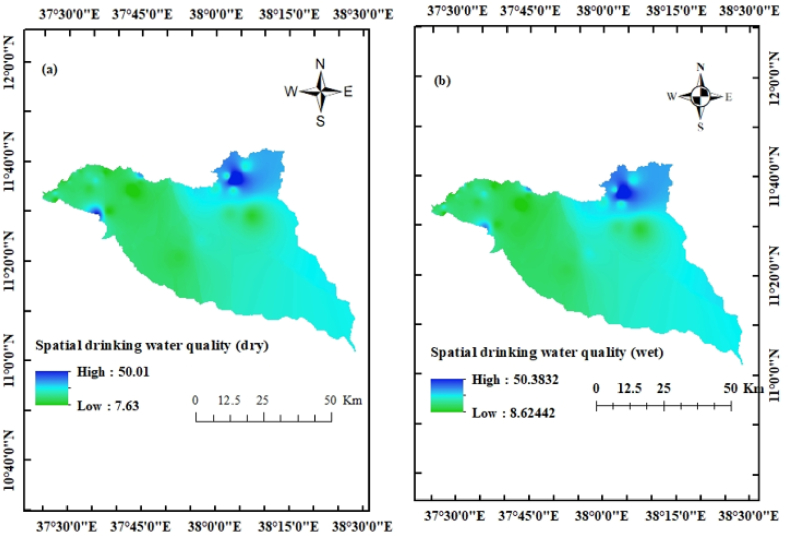
Drinking water quality status (a) dry season and (b) wet season.

EWQI is an advanced technique to evaluate the status of groundwater quality for domestic uses. The results revealed that 84.6% of the samples had excellent water quality, 12.8% (GW1, GW2, GW3, GW24, and GW28) had good water quality, and 2.6% (GW31) had medium water quality in both seasons (dry and wet) (Fig. 8 a and b). The GW31 has a medium water quality status, which is the lowest in comparison to the others. This is due to that the well is located in Este Town near the Wanka River, and the aquifer is shallow in nature. Due to poor water management practices, untreated water may interact with the river and enter directly. Good quality water is also found nearby Bahir Dar (GW1 and GW2), Arbgbya (GW24), Tisabay (GW3), and Mekane Eyesus (GW28 and GW31). This shows that the overall water quality of urban areas was lower than that of rural areas (urban areas are highly contaminated) [11]. This indicates that except for nitrate concentration, aquifers located near urban areas are more prone to groundwater pollution than rural aquifers. Therefore, urban areas and agricultural lands are the major sources of groundwater pollution [8].

### 4.5 Groundwater for irrigation purposes

Evaluation of water quality and application of proxy indices are essential in the study area because irrigation water quality is susceptible to chemical parameters [63]. Therefore, a graphical representation and eight indices were applied for a better understanding of the current groundwater situation as well as its suitability for agricultural activities (Fig. 4 a and b).

SAR is a common approach to evaluate irrigation water which relies on sodium concentration [70]. Thus, very high sodium concentration in the groundwater harms the soil and is unsuitable for plant productivity. During the analysis, it was found that 3% of samples have very high sodium water resulting in unsuitability for any crops. 10% of the total samples have medium sodium water (good), and 85% of samples have low sodium water (excellent). %Na is a measure of soil permeability in agricultural lands. Soil drainage permeability can reduce the higher sodium availability through irrigation water [71]. In the study, groundwater samples are categorized as excellent (18%), good (46%), medium (28%), and poor (8%). PS indicates the level of soluble salts in the water. PS values were classified as very high (69%), high (28%), and moderate (3%). The samples have moderate water and may have adverse effects on many crops. RSC results showed that groundwater samples (18%) were unsuitable with RSC values ≥ 2.5 meq/L and poor water quality was recorded in about 28% of samples. However, in 54% of the studied region, the groundwater was suitable regarding RSC values < 1.25. MAR values ranged from 0 to 68.43. Thirty locations (77% of samples) are suitable for irrigation regarding MAR values < 50% whereas nine locations (23% of samples) are not suitable for irrigation. The KI values varied from 0.08 to 11.34, and thirty (77%) samples are evaluated as suitable for irrigation (KI < 1). Three samples (8%) are considered unsuitable for irrigation (KI > 2). The remaining samples are marginal for irrigation uses. PI is one of the most important irrigation water quality indices to evaluate the long-term effects of soil permeability. Twelve locations of groundwater samples are suitable for irrigation and twenty-seven samples are considered marginal water for irrigation.

**Fig. 9:**
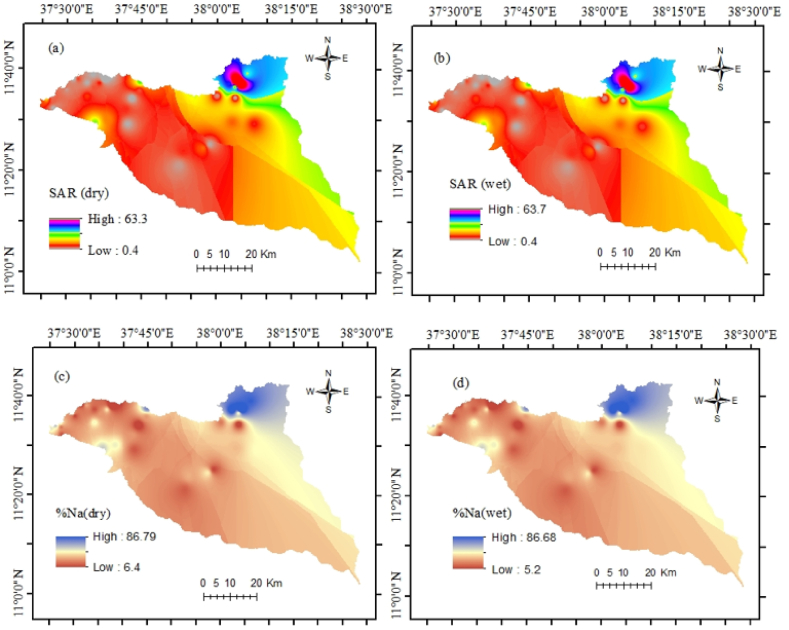

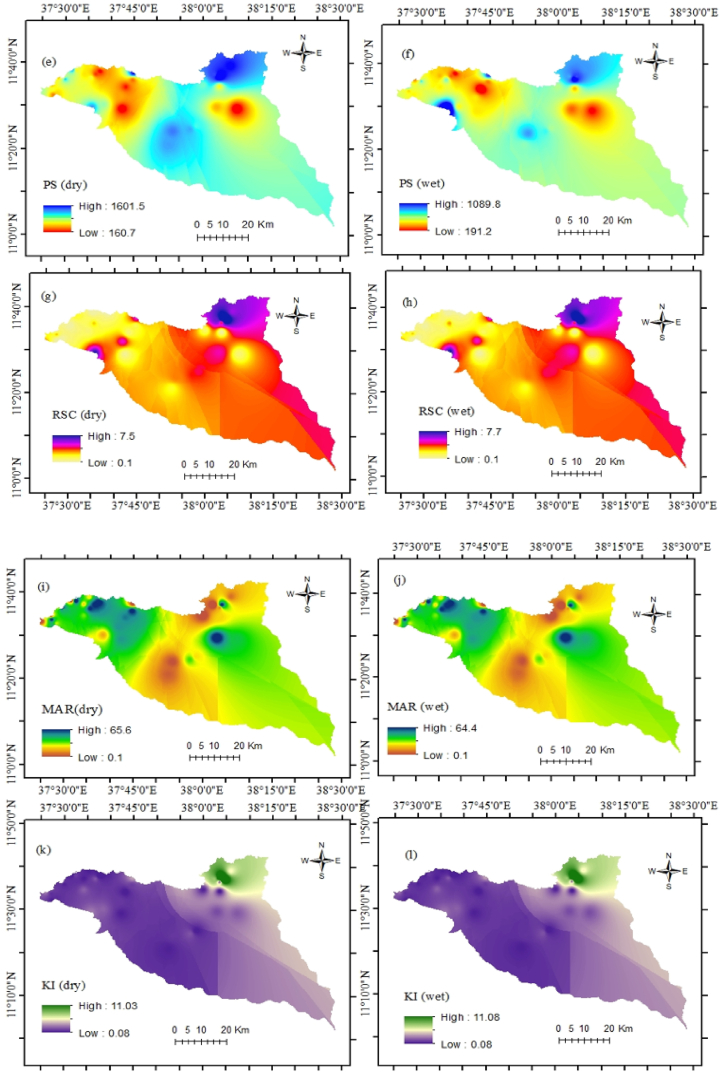

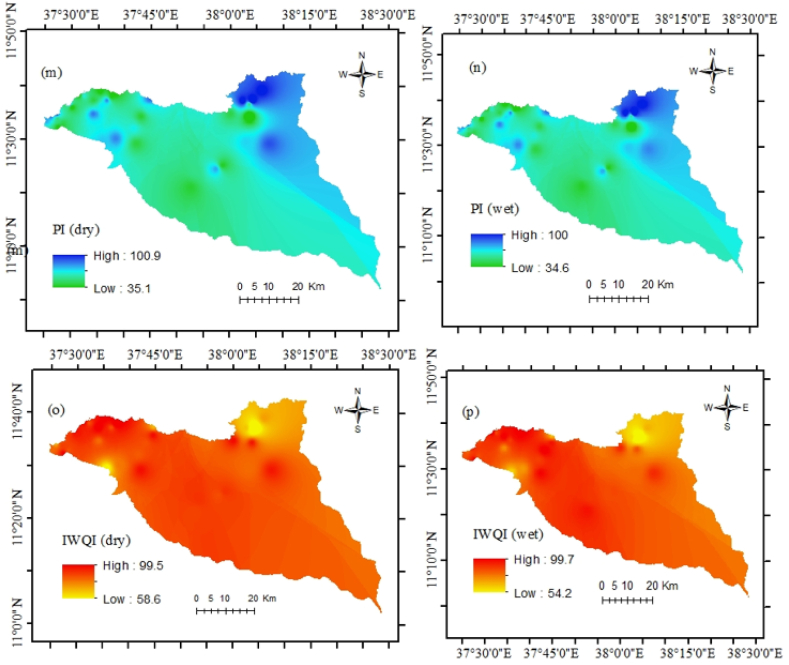
Irrigation water quality status (a) SAR dry, (b) SAR wet, (c) %Na dry, (d) %Na wet, (e) PS dry, (f) PS wet, (g) RSC dry, (h) RSC wet, (i) MAR dry, (j) MAR wet, (k) KI dry, (l) KI wet, (m) PI dry, (n) PI wet, (o) IWQI dry, and (p) IWQI wet.

According to the IWQI values, 28 water wells have no restriction on irrigating for any type of farming. 8 wells also represent low restriction, and the remaining 3 wells represent moderate quality. Generally, the seasonal spatial and temporal groundwater quality status for drinking and irrigation purposes has been figured out in both seasons (Fig. 8 a and b and Fig. 9 a - p).

Generally, sodium and salinity hazards have a potential to reduce the soil permeability (which restricts plant growth through reduction of water circulation in the soil) and soil salinity (which restricts root to intake salt), respectively [53].

### 4.6 Limitation of the study

Due to limitation of funding and laboratory access, this research work did not focus on heavy metals (trace elements) which linked with public health. Because heavy metals have a potentially harm for human and aquatic environment [72]. Boron concentration was not evaluated in the study area for irrigation purposes. However, the main physicochemical parameters were considered under the guideline of World Health Organization [51]. The next scholars should focus on trace elements. Moreover, suitable and simple groundwater management measure should be required to improve the crop yields and public health in study area.

## 5 Conclusions

In the study area, groundwater is the only source to meet the demand for drinking and irrigation purposes. Thus, it is mandatory to validate their water quality status before it enters the distribution system for drinking and irrigation purposes. The physicochemical parameters results were compared with modified WHO guidelines. The laboratory results showed that some nitrate concentration, TDS, EC, and pH values are not within the standard limits. The seasonal variation of physicochemical parameters between dry and wet was insignificant. However, shallow unconfined aquifer wells have some variation in the case of nitrate, pH, and EC. Based on proxy indices such as EWQI and SAR, %Na (ESP), MAR, KR, PI, RSC, PS, and IWQI), about 85% of the total samples are categorized as suitable water for drinking and irrigation purposes, but a few samples are affected by various geological and anthropogenic activities located near urban areas. The relative water quality status in a rural area is better than in the urban area, except for the contaminated level of nitrate. Excessive use of fertilizers by farmers in rural areas somewhat affects the quality. Hydrogeochemical results revealed that Ca–HCO3> Ca–Cl > Na–HCO3 are the most dominant groundwater types in the study area. Calcium carbonate water is found in shallow aquifers increasing the hardness. Sodium carbonate water is located in deep groundwater which is due to higher exchangeable rock-water interactions. These comprehensive evaluation techniques will provide a map-based groundwater quality status for planners, decision-makers, and water resources managers. More than 85% of samples are almost suitable for drinking and irrigation uses. Excessive application of fertilizers and improper wastewater disposal needs to be monitored on regular basis. Once the groundwater becomes contaminated, it is too difficult to treat. Therefore, monitoring of a large number of physicochemical parameters will be recommended to control the degree of groundwater contamination. Integrally, the model is a useful tool for water resources management for both environmentalists and public health decision-makers.

## Author contribution statement

Asnakew Mulualem Tegegne: Conceived and designed the experiments; Performed the experiments; Analyzed and interpreted the data; Contributed reagents, materials, analysis tools or data; Wrote the paper.

Tarun Kumar Lohani: Conceived and designed the experiments; Performed the experiments; Analyzed and interpreted the data; Wrote the paper.

Abunu Atlabachew Eshete: Conceived and designed the experiments; Performed the experiments; Analyzed and interpreted the data; Wrote the paper.

## Data availability statement

Data will be made available on request.

## Declaration of interest’s statement

The authors declare no conflict of interest.
